# Editorial

**DOI:** 10.1017/qrd.2020.3

**Published:** 2020-08-06

**Authors:** Bengt Norden

**Affiliations:** 1 Chair of the Board of Editors, QRB Discovery; 2 Chair of Physical Chemistry, Department of Chemistry and Chemical Engineering, Chalmers University of Technology, SE-41296 Gothenburg, Sweden

On the 51st anniversary of *Quarterly Reviews of Biophysics* (QRB) and on my 4th year as Editor-in-Chief, it is with pleasure that I announce the new open access journal from Cambridge University Press will provide an outlet for exciting new discoveries in the burgeoning field of biophysics.

The section called Discovery, which was tested in previous years as part of *Quarterly Reviews of Biophysics* (QRB), is now upgraded and relaunched as a journal on its own right. The launch of *QRB Discovery*, which coincided with the annual conference of the Biophysical Society, promises those working in the field a fast, transparent way to publish cutting edge results. The focus for *QRB Discovery* will be on biological phenomena that can be described and analysed from a molecular angle.

Biophysics applies approaches and methods traditionally used in physics and maths to study the living world, from molecules and cells right up to populations of animals and plants. This interdisciplinary approach has a huge number of applications and has the potential to address some of the biggest challenges facing our species and our planet. It is vital that discoveries with the potential to benefit society are published quickly and transparently.

The field has been missing a dedicated place to publish ground-breaking results – ‘discoveries’ – that point towards an exciting direction, rather than presenting of a traditional comprehensive study. This is the gap *QRB Discovery* will seek to fill.

Authors are encouraged to elaborate on the potential consequences and wider impact of their discoveries. If the research is of high quality and it is a sound result that points in an exciting direction – even if it is speculative – we will publish.

This transparency is further extended by publishing open peer review reports. This will, expectantly, promote a more constructive type of review for authors but it will also contribute to the recognition of reviewers.

I look forward to see what exciting research will be submitted next!

